# Risk of cognitive decline progression is associated to increased blood‐brain‐barrier permeability: A longitudinal study in a memory unit clinical cohort

**DOI:** 10.1002/alz.13433

**Published:** 2023-09-19

**Authors:** Albert Puig‐Pijoan, Joan Jimenez‐Balado, Aida Fernández‐Lebrero, Greta García‐Escobar, Irene Navalpotro‐Gómez, Jose Contador, Rosa‐María Manero‐Borràs, Victor Puente‐Periz, Antoni Suárez, Francisco J. Muñoz, Oriol Grau‐Rivera, Marc Suárez‐Calvet, Rafael de la Torre, Jaume Roquer, Angel Ois

**Affiliations:** ^1^ Department of Neurology Hospital Del Mar Barcelona Spain; ^2^ Hospital del Mar Medical Research Institute (IMIM) Barcelona Spain; ^3^ Medicine Department Universitat Autònoma de Barcelona Barcelona Spain; ^4^ ERA‐Net on Cardiovascular Diseases (ERA‐CVD) consortium Barcelona Spain; ^5^ RICORS‐ICTUS, Enfermedades Vasculares Cerebrales Instituto de Salud Carlos III Madrid Spain; ^6^ Barcelonaβeta Brain Research Center (BBRC) Pasqual Maragall Foundation Barcelona Spain; ^7^ Department of Medicine and Life Sciences Universitat Pompeu Fabra Barcelona Spain; ^8^ Centro de Investigación Biomédica en Red de Fragilidad y Envejecimiento Saludable (CIBERFES) Madrid Spain; ^9^ CIBER Fisiopatología Obesidad y Nutrición (CIBERObn) Instituto de Salud Carlos III Madrid Spain

**Keywords:** blood‐brain barrier, cerebrovascular disease, cognition, dementia, vascular dementia

## Abstract

**INTRODUCTION:**

This study examined the relationship between blood‐brain‐barrier permeability (BBBp), measured by cerebrospinal fluid/serum albumin ratio (QAlb), and cognitive decline progression in a clinical cohort.

**METHODS:**

This prospective observational study included 334 participants from the BIODEGMAR cohort. Cognitive decline progression was defined as an increase in Global Deterioration Scale and/or Clinical Dementia Rating scores. Associations between BBBp, demographics, and clinical factors were explored.

**RESULTS:**

Male sex, diabetes mellitus, and cerebrovascular burden were associated with increased log‐QAlb. Vascular cognitive impairment patients had the highest log‐QAlb levels. Among the 273 participants with valid follow‐up data, 154 (56.4%) showed cognitive decline progression. An 8% increase in the hazard of clinical worsening was observed for each 10% increase in log‐QAlb.

**DISCUSSION:**

These results suggest that increased BBBp in individuals with cognitive decline may contribute to clinical worsening, pointing to potential targeted therapies. QAlb could be a useful biomarker for identifying patients with a worse prognosis.

## 1 BACKGROUND

Cognitive impairment (CI) and dementia, including Alzheimer's disease (AD), are major causes of disability and dependency worldwide, affecting millions of people and their families.^1^ The impact of these conditions on quality of life is substantial and represents a significant public health challenge. The blood‐brain barrier (BBB) plays a crucial role in maintaining brain homeostasis and health by regulating the exchange of molecules into and out of the brain parenchyma.^2^ Disruption of the BBB may contribute to the development and progression of CI and dementia.^3^ Vascular risk factors and cerebrovascular pathology are common in all stages of CI and dementia and have been shown to affect BBB integrity.^4^, ^5^, ^6^ Previous studies reported an increase in BBB permeability (BBBp) in individuals with CI.^3^, ^7^, ^8^, ^9^ Moreover, cerebrovascular pathology and BBB dysfunction have been linked to AD pathophysiology.^10^, ^11^ The cerebrospinal fluid (CSF)/serum albumin ratio (QAlb) is a widely used parameter for measuring BBBp with respect to large molecules.^12^, ^13^, ^14^ Due to its reproducibility and relative ease of determination in clinical laboratories, it may be useful in studies of cognitive decline and other neurological diseases.^12^, ^15^, ^16^, ^17^, ^18^, ^19^, ^20^ However, its clinical utility has not been well established.

The primary objective of this study was to assess the association between BBBp (as measured by QAlb) and the risk of progression of cognitive decline in a clinical cohort of individuals with cognitive decline at different stages, from subjective cognitive decline to severe dementia. Our secondary objective was to explore the independent relationship between BBBp and demographic, clinical, vascular, etiologic, and radiological factors.

By investigating the relationship between BBBp and cognitive decline progression in a real‐world clinical cohort, this study aimed to increase knowledge on the underlying mechanisms of CI and dementia and to identify potential biomarkers that may be useful in patient management.

**RESEARCH IN CONTEXT**

1. **Systematic review**: The authors conducted a comprehensive literature review using traditional sources (eg, PubMed) to explore blood‐brain‐barrier permeability (BBBp) and albumin quotient (QAlb) in cognitive decline. Despite numerous publications on this topic, few have investigated their significance in real‐world memory clinic populations. Relevant publications are cited throughout the manuscript.
2. **Interpretation**: In this longitudinal observational study in a memory clinic population, we found that increased BBBp was associated with cognitive decline progression. This suggests that QAlb could serve as a potential biomarker for identifying individuals at higher risk of cognitive decline progression and that increased BBBp may be linked to clinical worsening.
3. **Future directions**: Additional research should focus on understanding the underlying mechanisms of increased BBBp and developing effective therapeutic strategies targeting BBB dysfunction in patients with cognitive decline. Future studies should investigate the potential role of QAlb as a prognostic biomarker in clinical settings, which could lead to improved patient assessment and management.

## 2 METHODS

### 2.1 Participants and study design

We conducted a prospective observational study of patients consecutively included in the BIODEGMAR cohort^21^ between September 2017 and September 2021. The BIODEGMAR is an observational longitudinal study that enrolls individuals with memory complaints or cognitive decline admitted at the Cognitive Decline and Movement Disorders Unit, Department of Neurology, Hospital del Mar (Barcelona, Spain). As a clinical cohort of a memory unit, the BIODEGMAR cohort includes participants with a high heterogeneity in demographics, comorbidities, and disease presentations, reflecting a real‐world scenario. *Inclusion criteria*: (i) subjects evaluated in the Cognitive Decline and Movement Disorders Unit at Hospital del Mar included in DEGMAR register (see eMethods in Supplement 1); (ii) signed informed consent; (iii) meet diagnostic criteria for the following syndromes and conditions (see ‘clinical diagnostic’). *Exclusion criteria*: (i) ≥80 years old; (ii) any contraindication for lumbar puncture; (iii) do not agree with study procedures. The procedures of the BIODEGMAR study include a clinical visit, an extensive neuropsychological evaluation, magnetic resonance imaging (MRI), *APOE* genotyping, a lumbar puncture for CSF collection, and blood sampling.^22^ A comprehensive description of the BIODEGMAR cohort, the inclusion and exclusion criteria, study procedures, the core AD CSF biomarkers measurements, and cutoffs determination can be found in the eMethods in the Supplement 1.

### 2.2 Sociodemographic and clinical data

The sociodemographic and clinical information was collected using an extensive structured questionnaire.^21^ Briefly, we collected sociodemographic information including sex, age, years of education, and vascular risk factors such as hypertension, diabetes mellitus type 2 (DM), hyperlipidemia, and active smoking habit.

### 2.3 Clinical diagnosis

Participants in the BIODEGMAR cohort with a Global Deterioration Scale (GDS) > 1^22^ and the following clinical diagnosis were included in the current study: subjective cognitive complaints (*n* = 21)^23^; mild cognitive impairment syndrome (MCI; *n* = 110)^24^; AD‐type dementia (possible, probable, and atypical presentation; *n* = 120)^25^; behavioral variant Frontotemporal Dementia (*n* = 12)^26^; primary progressive or progressive aphasia (logopenic, progressive non fluent, and semantic variants; *n* = 17)^27^; Lewy body dementia (LBD; *n* = 9)^28^; corticobasal syndrome (CBS)^29^ and progressive supranuclear palsy (PSP) (*n* = 8)^30^; vascular cognitive impairment and dementia (VCID; *n* = 12)^31^; and cerebral amyloid angiopathy (CAA; *n* = 11).^32^ Individuals with other causes of dementia or with an unspecified clinical diagnosis were included as “other” (*n* = 9).

### 2.4 Core AD CSF biomarkers

Core AD CSF biomarkers (Aβ42/40, p‐tau181 and t‐tau) were measured at the Laboratori de Referència de Catalunya with Lumipulse, Fujirebio.^21^ AD was biologically defined (b‐AD) according to CSF amyloid beta (Aβ) 42/p‐tau181 ratio < 10.25,^33^ regardless of clinical diagnostic. Extensive information about core CSF AD biomarkers and cutoffs are included in eMethods Supplement 1.

### 2.5 QAlb (CSF/serum albumin)

Serum albumin was measured by colorimetric method using bromocresol green. CSF albumin was measured by the immunoturbidimetry method. Both serum and CSF albumin measurements were performed with Cobas‐Hitachi automated reagents and systems (Roche Diagnostics GmbH). Higher levels of QAlb point to increased BBBp.^13^

### 2.6 Neuroimaging and cerebrovascular burden (CVB)

Brain MRI was performed in all participants, except in cases with contraindication (eg, pacemaker, MRI‐incompatible aneurysm clips, and claustrophobia). In case of contraindication for MRI, head CT scan was performed as part of the clinical diagnostic process. Structural MRI scans were acquired on 1.5 T (General Electric Signa Explorer) or 3T (Philips Achieva). The imaging protocol included T1‐ and T2‐weighted sequences, high‐resolution T1 3D, diffusion‐weighted images, FLAIR imaging, gradient echo, and/or ven‐Bold sequences. CVB was defined as the presence of extensive white matter hyperintensities as a subcortical and/or periventricular Fazekas’ score^34^ higher than 1 and/or presence of any brain vascular infarct or hemorrhagic lesion including microbleeds.^35^

### 2.7 Follow‐up and neuropsychological controls

A clinical visit after lumbar puncture was performed (2 to 8 weeks). Follow‐up visits, including clinical and neuropsychological evaluations, were conducted yearly (12 ± 2 months). According to clinical and neuropsychological evaluations, Global Deterioration Scale (GDS) and Clinical Dementia Rating (CDR; global score)^36^ were assessed to determine the cognitive decline stage. The endpoint for longitudinal analyses was progression of cognitive decline, which was defined as a binary variable, with patients undergoing any increase in GDS and/or CDR scales during the follow‐up being considered as progressors and those without increase in these scales as nonprogressors. Other clinical visits were performed at a neurologist's discretion depending on clinical necessities. Clinical records were reviewed to check on the possible progression of cognitive decline before annual visits. The present study followed up patients until September 2022. We only included those subjects with GDS and CDR scores lower than 6 and 3, respectively, where a GDS score of 6 indicates a severe dementia stage and a CDR of 3 is the highest possible. We defined a minimum time of follow‐up of 12 months. All cases were reviewed at the conclusion of follow‐up to confirm cognitive decline progression and rule out fluctuations of symptoms (*n* = 5).

### 2.8 Standard protocol approvals, registrations, and patient consent

The DEGMAR register and the BIODEGMAR study were approved by the Drug Research Ethics Committee (CEIm), Barcelona (CEIm PSMAR, project code 2018/7805I). All participants (and/or a representative when appropriate), provided written informed consent approved by our local ethics committee (CEIm PSMAR).

### 2.9 Statistical analysis

#### 2.9.1 Sample characteristics

**FIGURE 1:**
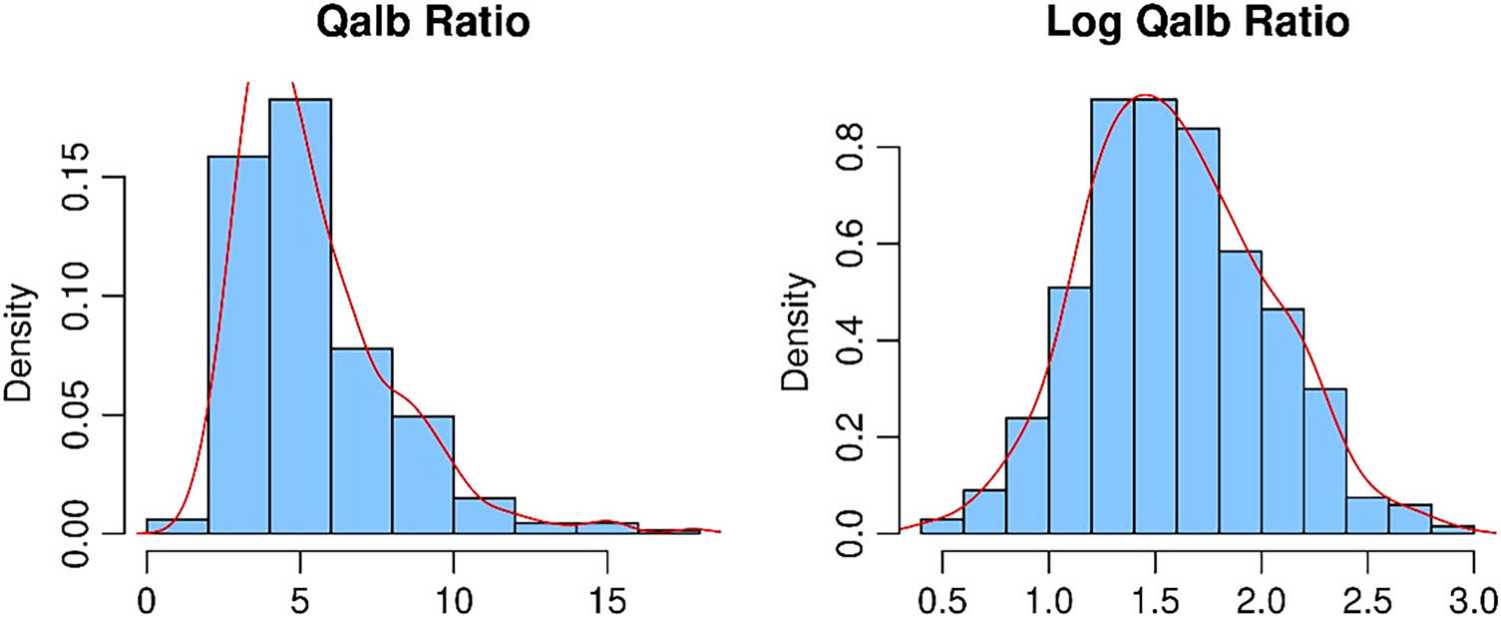
Distribution of Qalb levels before (left) and after (right) log_e_ transformation.

In the descriptive analyses, we present data as medians (interquartile range) for continuous variables and as frequencies (percentage) for categorical variables. As we show in Figure 1, QAlb levels had a left‐skewed distribution, and we applied the log_e_ transform, which is used henceforth as the variable of interest (log‐QAlb).

#### 2.9.2 Cross‐sectional association between BBBp and main clinical factors

We first conducted a series of univariate analyses to assess which clinical factors or diagnoses were associated with QAlb at the baseline visit (cross‐sectional analysis, *N* = 334). To that end, we used Spearman's correlation, *t*, or ANOVA tests, as appropriate.

#### 2.9.3 Longitudinal effect of BBBp on risk of cognitive decline progression

In the second part of the analysis, we focused on a subset of patients with valid follow‐up data (longitudinal analysis, *N* = 273). These patients were followed up until September 2022, and we registered the incidence of cognitive decline progression. Longitudinal data were right censored at the third year of follow‐up to avoid an excessive number of censored patients. We first used Kaplan‐Meier curves to observe which clinical variables were associated with the progression of cognitive decline within the follow‐up, and significance levels were estimated using the log‐rank test. The cognitive decline progression rate is presented as 1 (survival function) and 95% confidence intervals (CIs). For the univariate analysis, continuous variables were split into tertiles (post hoc comparisons were corrected for family‐wise error with Bonferroni adjustment). We subsequently built multivariate Cox regression models to estimate the effect of QAlb on clinical progression after adjusting for potential confounders that were selected according to the univariate analysis (*p* ≤ .1) or previous literature. Therefore, the scope model was fully adjusted for age, sex, education, hypertension, diabetes, hyperlipidemia, b‐AD, CDR scale, cerebrovascular burden, and log‐QAlb. Variables were selected via a forward stepwise algorithm, and the metric of interest was the Akaike information criterion. We chose to adjust this multivariate model only for the baseline CDR instead of both the CDR and GDS scores to avoid having to deal with collineary. For the last model, we checked Schoenfeld and deviance residuals to detect potential violations, discard outliers, and confirm the proportional hazard assumption. We set the α value at 0.05. A team of two biostatisticians reviewed all statistical analyses (JJ‐B, A.O.). All the statistical analyses were done with R software (R version 4.2.1; Copyright (c) 2022 The R Foundation for Statistical Computing, Vienna, Austria).

## 3 RESULTS

### 3.1 Participant characteristics

From September 22, 2017 to September 29, 2021, a total of 360 individuals were included in the BIODEGMAR cohort, 350 with GDS > 1. For this study, we excluded 11 participants with undetermined plasma or CSF albumin levels, three cases of rapidly progressive dementia caused by Creutzfeldt‐Jakob disease (CJD), one case with a disseminated neoplastic disease, and one case with an active systemic inflammatory disease.

### 3.2 Sample characteristics

Table 1 shows the main demographic, clinical, and radiological characteristics of the sample (*N* = 334); the median age was 74 years (Q_1_‐Q_3_: 69 to 77), and 195 (58.4%) were females. Median QAlb was 4.75 mg/g (Q_1_‐Q3: 3.66 to 6.58).

**TABLE 1:** Main characteristics of sample (*N* = 334).

Age	74.0 [69.0; 77.0]
Sex, Female	195 (58.4%)
Education, years	8.00 [6.00; 10.0]
Hypertension	186 (55.7%)
Diabetes mellitus	72 (21.6%)
Hyperlipidemia	167 (50.0%)
Active smoker	28 (9.36%)
Cerebrovascular burden	121 (38.2%)
b‐AD	217 (65.0%)
*ApoE*‐ε4 carrier	114 (45.8%)
GDS score	
2	27 (8.08%)
3	128 (38.3%)
4	125 (37.4%)
5	41 (12.3%)
6	13 (3.89%)
CDR score	
0	26 (7.78%)
0.5	127 (38.0%)
1	86 (25.7%)
2	78 (23.4%)
3	17 (5.09%)

*Note*: Values represent medians (interquartile range) or numbers (percentage) according to type of each variable. *Missing data*: education, five cases; smoking, 35 cases; cerebrovascular burden, 17 cases; *ApoE* polymorphism: 85 cases.

Abbreviations: b‐AD, biologically defined AD (according to abnormal CSF Aβ42/p‐Tau181 ratio; CDR, clinical dementia rating scale; GDS, global deterioration scale.

### 3.3 Cross‐sectional association between BBBp and main clinical factors

**FIGURE 2:**
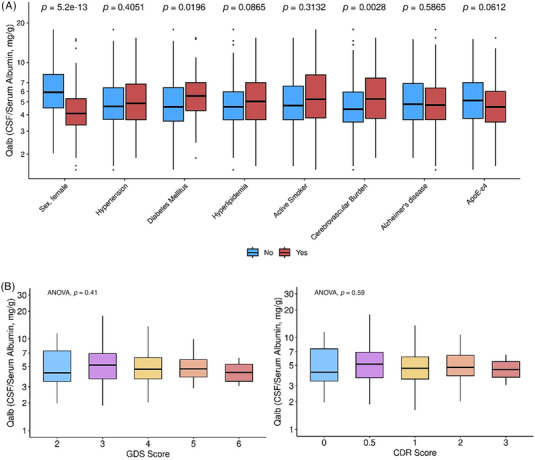
Cross‐sectional differences in levels of Qalb for main clinical variables. (A) Differences in Qalb levels for main vascular and cognitive impairment risk factors (*p* values represent pairwise *t* test contrasts). (B) Illustration of how Qalb differs according to clinical stage both for GDS and CDR scales; *p* values were obtained with ANOVA tests. In both panels, the *y* axis (Qalb levels) were log‐scaled.

**FIGURE 3:**
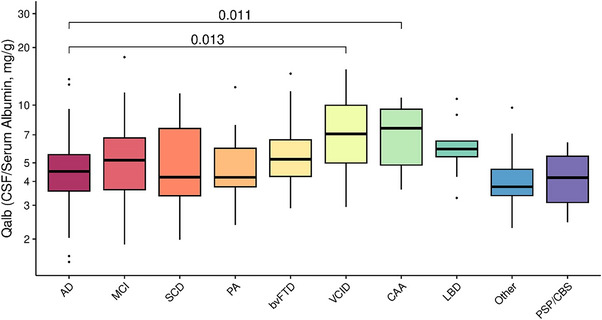
Qalb levels by clinical diagnosis. Groups were compared with pairwise *t* tests; square brackets indicate which groups are significantly different at *p* < 0.05. AD, Alzheimer's disease; bvFTD, behavioral variant frontotemporal dementia; LBD, Lewy body dementia; MCI, mild cognitive impairment; PA, progressive aphasia; PSP/CBS, progressive supranuclear palsy and/or corticobasal syndrome; SCD, subjective cognitive complaints; VCID, vascular cognitive impairment and dementia.

We first analyzed which of the clinical variables included in Table 1 were associated with log‐QAlb levels and found that males presented higher permeability levels than females (Figure 2A). Similarly, the presence of DM and CVB was associated with increased log‐QAlb (Figure 2A). On the other hand, clinical staging according to GDS or CDR did not affect log‐QAlb levels (Figure 2B). Neither age (*r* = 0.02, *p* = 0.655) nor years of education (*r* = 0.06, *p* = 0.250) were significantly correlated with BBBp as measured by log‐QAlb. We finally compared how log‐QAlb levels differed between subjects according to their clinical diagnosis, finding global significant differences across groups (F (9324) = 2.89, *p* = 0.003). Post hoc comparisons revealed that VCID patients had the highest levels of log‐QAlb and were significantly different compared to the AD‐type dementia group, which presented the lowest levels of log‐QAlb together with the progressive aphasia group (Figure 3).

### 3.4 Longitudinal effect of BBBp on risk of cognitive decline progression

Among 334 cases, we followed up with 273 (81.7%) patients within 3 years (median follow‐up [Q_1_ to Q_3_]: 17 months [12.5 to 29]), representing 5675 patient‐months. A total of 61 participants were excluded from this analysis, 48 participants because they did not have a minimum follow‐up of 12 months and 13 participants due to advanced dementia stage at baseline. Twenty‐nine participants were censored due to loss of follow‐up (*n* = 18) or death (*n* = 11).

**FIGURE 4:**
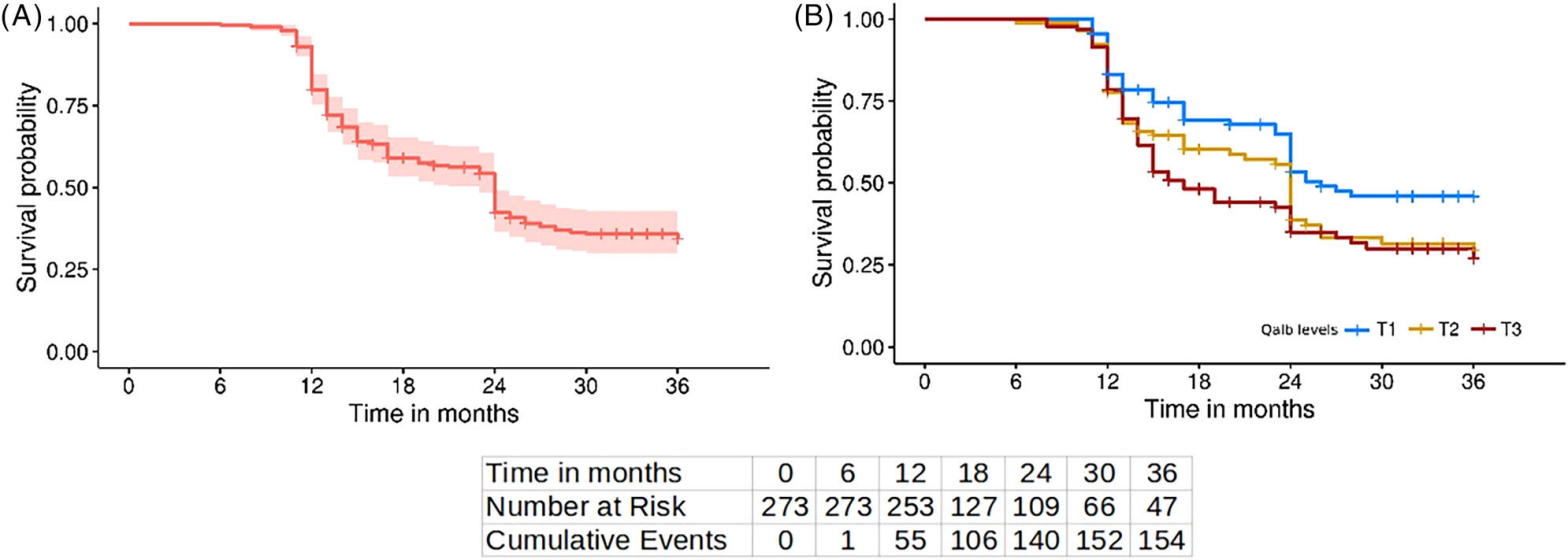
Survival curve of sample. Both survival curves show likelihood of cognitive decline progression within follow‐up, as an increment in GFS and/or CDR scales. (A) Survival curve of sample, where light red shade represents 95% confidence intervals, and vertical lines are censored patients. (B) Same survival curve by Qalb levels, split into tertiles (T1, blue; T2, yellow; and T3, red). At bottom of both figures are the number of patients at risk of progression at the beginning of each time point and the cumulative number of events at the end of the same time point.

We detected 154 (56.4%) patients showing progression of cognitive decline, which represents an event rate of 65.7% in the whole sample at the end of the follow‐up (95% CI: 58.6% to 71.7%, Figure 4A). In Table 2 we show the effect of the main clinical variables on the risk of cognitive decline progression. As we visually represent in Figure 4B, patients with higher BBBp presented an increased rate of progression at the end of the follow‐up (log‐rank *p* value = .020). In post hoc pairwise comparisons, we found that this result was driven by patients with log‐QAlb ≤ 3.61 (first tertile: cognitive decline progression rate at 3 years = 54%) as compared to patients with log‐QAlb > 6.62 (third tertile: cognitive decline progression rate at 3 years = 73%, log‐rank *p* value_Bonferroni_ = .02). An AD *core* biomarker profile in CSF (b‐AD), *ApoE‐ε4* carrier status, and higher GDS or CDR score were all associated with progression of cognitive decline as well (Table 2).

**TABLE 2:** Effect of main clinical variables and Qalb on risk of clinical decline progression (*N* = 273).

Variable	CDP rate (95% CI)	*P*	Variable	CDP rate (95% CI)	*P*
*Age, years*			*QAlb*, mg/g		
Tertile 1 [50, 69]	0.56 (0.45;0.67)	0.1599	Tertile 1 [1.52, 3.61]	0.54 (0.43;0.65)	0.0199
Tertile 2 [70, 77]	0.73 (0.63;0.83)		Tertile 2 [3.61, 6.62]	0.71 (0.6;0.82)	
Tertile 3 [78, 84]	0.69 (0.56;0.82)		Tertile 3 [6.62, 17.80]	0.73 (0.62;0.84)	
*Sex, female*			*GDS score*		
Yes	0.65 (0.55;0.75)	0.3338	2	0.19 (0.02;0.36)	<0.0001
No	0.67 (0.59;0.75)		3	0.62 (0.52;0.72)	
*Education, years*			4	0.77 (0.67;0.87)	
Tertile 1 [0, 6]	0.64 (0.53;0.75)	0.2833	5	0.84 (0.7;0.98)	
Tertile 2 [7, 10]	0.76 (0.66;0.86)		*CDR score*		
Tertile 3 [11, 20]	0.56 (0.44;0.68)		0	0.2 (0.02;0.38)	0.0001
*Hypertension*			0.5	0.62 (0.52;0.72)	
Yes	0.66 (0.57:0.75)	0.7579	1	0.76 (0.65;0.87)	
No	0.65 (0.55;0.75)		2	0.8 (0.67;0.93)	
*Diabetes*			*CVB*		
Yes	0.73 (0.6;0.86)	0.2950	Yes	0.76 (0.66;0.86)	0.1217
No	0.63 (0.56;0.7)		No	0.64 (0.55;0.73)	
*Hyperlipidemia*			*b‐AD*		
Yes	0.71 (0.62;0.8)	0.3878	Yes	0.8 (0.73;0.87)	<0.0001
No	0.6 (0.5;0.7)		No	0.38 (0.27;0.49)	
*Active smoker*			*ApoE‐ε4*		
Yes	0.7 (0.51;0.89)	0.1535	Yes	0.77 (0.68;0.86)	0.0008
No	0.66 (0.59;0.73)		No	0.51 (0.41;0.61)	

*Note*: Values represent rate of clinical progression at third year of follow‐up according to Kaplan‐Meier estimator (1 − survival rate) and 95% confidence intervals for each variable and group. Significance levels were calculated using log‐rank test. Continuous variables (Qalb, age, and education) were split into tertiles. Missing data: education, three cases; smoking, 25 cases; cerebrovascular burden, 14 cases; ApoE polymorphism, 61 cases.

Abbreviations: b‐AD, biologically defined AD (according to abnormal CSF Aβ42/p‐Tau181 ratio); CI, confidence interval; CDP, clinical decline progression; CDR, clinical dementia rating scale; CVB, cerebrovascular burden; CDR, clinical dementia rating scale; log‐QAlb, log‐scaled CSF/serum albumin ratio.

After adjusting for variables that were associated with clinical worsening in the univariate analysis or that have been described to increase the risk of cognitive decline (age, sex, years of education, hypertension, diabetes, hyperlipidemia, b‐AD, CDR scale, and CVB), we analyzed the independent effect of BBBp on this progression. Variables were selected for inclusion in the final regression model using a forward stepwise algorithm, and only sex, b‐AD, CDR, and log‐QAlb were found to be significant predictors of cognitive decline progression in our cohort. As we show in Table 3, for each 10% increase in log‐QAlb levels we observed an 8% increase in the hazard of clinical worsening (95% CI = 1.04 to 1.13, *p* < 0.001). Similarly, female sex was associated with increased risk of cognitive decline progression such that females showed a 1.63‐fold increased hazard of progression at the end of the follow‐up as compared to males (Table 3).

**TABLE 3:** Cox regression models showing effect of BBBp on risk of cognitive decline progression (*N* = 273).

	HR (95% CI)	*P*
Sex, female	1.63 (1.12;2.36)	0.0099
Log‐QAlb, 10% increase in Qalb	1.08 (1.04;1.13)	0.0003
Alzheimer's disease	2.09 (1.39;3.13)	0.0004
CDR Score = 0.5	2.92 (1.05;8.16)	0.0408
CDR Score = 1	3.23 (1.13;9.21)	0.0286
CDR Score = 2	4.67 (1.62;13.47)	0.0043

*Note*: The dependent variable was the incidence of CDP within 3 years, and values represent hazard ratios, 95% confidence intervals, and *p* values. Variables were selected via a forward stepwise algorithm, where the metric of interest was the Akaike information criterion and the scope model was fully adjusted for age, sex, education, hypertension, diabetes, hyperlipidemia, Alzheimer's disease, CDR scale, cerebrovascular burden, and log‐Qalb. In this table we show the final model at the last iteration.

Abbreviations: BBBp, blood‐brain‐barrier permeability; CI, confidence interval; CDR, clinical dementia rating scale; HR, hazard ratio; log‐QAlb, log‐scaled CSF/serum albumin ratio.

## 4 DISCUSSION

In our study, we found that QAlb was positively associated with male sex, DM, and CVB. We did not observe any association with AD CSF profile, nor with CI severity. Notably, we found that increased BBBp was independently associated with progression of cognitive decline.

### 4.1 Cross‐sectional association between BBBp and main clinical factors

Associations between QAlb, male sex,^20^, ^37^, ^38^ and vascular risk factors were previously described.^18^, ^19^, ^20^, ^39^, ^40^ In our study, although only the association of log‐QAlb with DM reached statistical significance, QAlb was positively associated with all vascular risk factors (HTA, DM, hyperlipidemia, and being active smoker). Moreover, patients with CVB showed higher QAlb levels, in line with previous studies.^39^, ^40^ In addition, we found higher Qalb levels in patients with a clinical diagnosis of VCID, as previously reported in similar studies.^16^, ^17^, ^39^

On the other hand, clinical staging according to GDS or CDR was not associated with QAlb, neither age nor b‐AD. To the best of our knowledge, no recent studies evaluated BBBp and CI syndrome caused by diverse etiologies at several stages, as we did in this study. Although most participants were classified as being between prodromal and mild to moderate dementia stages, observing a lower number of participants with subjective cognitive complaints (SCD) or advanced dementia stages (27 GDS 2, 13 GDS6), we find these numbers sufficiently robust. Our results might suggest that increased BBBp reaches a limit in mild dementia stages. However, it is important to point out that clinical diagnoses at each functional stage are diverse. Thus, we cannot rule out a relationship between QAlb and GDS or CDR scores within each specific group. Previous works showed a positive association of QAlb with aging.^38^, ^41^ In our study, we found no association, probably because of a relative short range of age distribution in our sample, which included patients visiting at a memory unit with a low number of young patients. We found no association between biologically defined AD (as AD CSF profile regardless of clinical diagnosis) and QAlb. Previous studies described altered BBBp in AD patients, from preclinical to dementia stages.^41^ These findings were consistent with results obtained in animal models that proposed amyloidosis as a potential factor contributing to increased BBBp.^8^, ^42^ However, recent studies do not support this hypothesis when BBBp is measured through determination of QAlb, which indicates increased BBBp to large molecules. A meta‐analysis by Olson et al.^12^ suggested that there was no significant change in QAlb levels in AD compared to other neurological diseases.^15^ Three additional studies^16^, ^18^, ^19^ reported that this association was more likely related to the well‐known coexistence of AD and vascular risk factors rather than being an AD‐specific pathophysiology. Our results support these findings, although we cannot rule out an association of increased BBBp and AD pathophysiology within the AD continuum, as our cohort did not include a cognitively unimpaired population.

### 4.2 Longitudinal data and associations of BBBp with increased risk of cognitive decline progression

Multiple studies have analyzed possible predictors that identify subjects at higher risk of clinical worsening, most of them in the AD continuum assessing AD pathology‐related fluid biomarkers as well as imaging biomarkers such as the dementia conversion‐related pattern (ADCRP) on [18F] Fluorodeoxyglucose Positron Emission Tomography (FDG PET).^43^, ^44^, ^45^, ^46^, ^47^ Our results show factors already described as strongly associated with progression of cognitive decline such as b‐AD (abnormal Aβ42/p‐Tau181 ratio), *APOE‐ε4* carrier status, and female sex.^48^ Abnormal levels of *core* AD CSF biomarkers are related to a higher risk of progression to dementia among SCD and MCI patients.^49^, ^50^, ^51^, ^52^, ^53^, ^54^, ^55^, ^56^ Interestingly, our results show an association between female sex and worse clinical prognosis, in line with previous evidence,^57^ although this effect might be modulated by sociodemographic aspects, suggesting a role of modifiable risk factors.^57^ Despite the growing interest in sex differences in cognitive decline, particularly in the AD continuum, there is a need for further epidemiological and clinical data to achieve a deeper understanding of this topic. Finally, our data showed a higher risk of cognitive decline progression as CDR increased. In recent years, few studies have compared the risk of clinical progression across different stages of cognitive impairment. A recent study^58^ proposed a model of disease progression in the AD continuum, although it did not include the progression risk within dementia stages. A previous work showed that the risk of institutionalization and death increases with age and severity stage.^59^ In our opinion, a better knowledge of prognostic factors during dementia stages of cognitive decline is needed, as patient management and the need of support change notably.

The results of our study show that increased BBBp, which is associated with vascular risk factors and CVB, is independently associated with a worse prognosis in individuals with cognitive decline. QAlb has been associated with a poorer prognosis in other neurological diseases such as spinal amyotrophic lateral sclerosis or multiple sclerosis.^60^, ^61^ To the best of our knowledge, this is the first time that QAlb has been related to the progression of cognitive decline. Moreover, the association between increased BBBp and clinical progression was observed independently of b‐AD. These results highlight the role of vascular pathophysiological mechanisms in neurodegenerative, vascular, and mixed etiologies of cognitive impairment.^4^, ^10^, ^11^ Of note, other fluid and imaging biomarkers of BBB alterations have provided valuable insights into the contributions of vascular dysfunction to cognitive decline and AD in recent years. Dynamic contrast‐enhanced MRI (DCE‐MRI) provides a method to estimate the index of BBB permeability (K^trans^) at the voxel level.^9^ Using this technique, previous studies found that BBB disruption started at the early stages of the disease in AD‐signature regions of brain such as the hippocampus and the parahippocampal gyrus,^9^, ^62^ finding differences between patients with a CDR of 0 and 0.5 independently of Tau and Aβ markers. Moreover, the soluble platelet‐derived growth factor β (sPDGFRβ), which is a marker of pericyte injury,^9^, ^63^, ^64^, ^65^, ^66^ is increased during the AD continuum, including early stages,^64^ and correlates with age and BBB breakdown as measured by K^trans^ and Qalb.^63^, ^67^ Interestingly, patients with progressive MCI show higher CSF sPDGFRβ levels as compared to non‐progressors.^63^ Moreover, baseline levels of sPDGFRβ in *APOE‐ε4* carriers predicted future cognitive decline after controlling for Aβ and Tau status.^68^ Altogether, Qalb, K^trans^, and sPDGFRβ might represent different aspects of physiopathological mechanisms involved in BBB disruption, which encourages study of their complementarity as markers of vascular dysfunction in cognitive impairment.

Our results may have important implications for the clinical management of patients with cognitive decline. Given the paradigm shift that the arrival of disease‐modifying therapies may entail soon, novel biomarkers for a better characterization of patients are needed. The AT(N) system^69^ might incorporate novel biomarkers of other pathophysiological mechanisms such as synaptic loss, neuroimmune dysregulation, or BBB dysfunction,^62^, ^70^ evolving toward an ATX(N) system^69^, ^71^ in which vascular dysfunction would be represented as ‘V.’^11^, ^71^ Our findings point to the possible use of QAlb as a vascular biomarker of BBB alteration in the ATV(N) system,^71^ providing important clinical insight on this regard besides a better characterization.^62^, ^70^ In individuals in the AD continuum, it might identify those individuals at higher risk of a more aggressive disease course. In addition to this, QAlb might serve as a prognostic biomarker among other neurodegenerative, vascular, and mixed etiologies beyond the AD continuum. Identifying AD‐negative individuals at high risk of clinical worsening through this marker holds clinical relevance, as there is a lack of prognostic biomarkers in these groups of patients. However, further studies focusing on the potential role of Qalb as a prognostic biomarker in other more homogeneous clinical cohorts with a larger number of patients is needed.

Our study is not free of limitations. First, as an observational study, our results do not establish causality, and the possibility of confounding factors cannot be ruled out. Additionally, as our sample size was relatively small, it would be important to investigate whether our findings are generalizable to other populations. It is also important to note that our sample consisted primarily of individuals with clinical diagnoses within the AD continuum, and the relatively limited number of participants with other diagnoses may limit the generalizability of our results. However, our cohort reflects the characteristics of a real‐world clinical memory unit, with a higher heterogeneity of demographic, comorbidities, and clinical presentations compared to research cohorts.

In conclusion, our study highlights the potential clinical utility of QAlb for identifying individuals at higher risk of progression of cognitive decline, suggesting that increased BBBp may contribute to clinical worsening and represent a potential therapeutic target. Further studies are needed to explore the underlying mechanisms of increased BBBp and to develop effective therapeutic strategies targeting BBB dysfunction in patients with CI.

## CONFLICT OF INTEREST STATEMENT

A.P.‐P. has served on advisory boards for Schwabe Farma Iberica. M.S‐C. has served as a consultant and on advisory boards for Roche Diagnostics International Ltd. and has given lectures at symposia sponsored by Roche Diagnostics, S.L.U. and Roche Farma, S.A. All other coauthors declare no competing interests. Author disclosures are available in the supporting information.

## CONSENT STATEMENT

All human subject participants in the study provided informed consent.

## Supporting information

Supporting information

Supporting information
