# Supplementary material for: Risk of cognitive decline progression is associated to increased blood‐brain‐barrier permeability: A longitudinal study in a memory unit clinical cohort
Source: Alzheimers Dement. 2023 Sep 19;20(1):538–48. doi: 10.1002/alz.13433 (PMC10916969; doi:10.1002/alz.13433)
Supplement: Supplementary file 1 — Supporting information [file ALZ-20-538-s001.docx]

**EMethods Supplement 1**

***Study participants, the DEGMAR register and the BIODEGMAR study.***

The clinical information, plasma and CSF samples of this study were collected from the DEGMAR register and the BIODEGMAR cohort study. The DEGMAR is an observational prospective register and database that includes all individuals visited in the Cognitive Decline and Movement Disorders Unit of Hospital del Mar in Barcelona, the reference unit in charge of cognitive disorders for a population of 350,000 inhabitants in the city. The DEGMAR register includes sociodemographic, clinical, neuropsychological and neuroimaging data. The BIODEGMAR cohort is an observational longitudinal study of patients included in the DEGMAR register. The procedures of the BIODEGMAR study include extensive neuropsychological evaluation, MRI, *APOE* genotyping, lumbar puncture for CSF collection and blood sampling. Follow-up visits, including neuropsychological and clinical evaluation, are performed once a year during the first three years after inclusion.

BIODEGMAR study procedures:

*Clinical evaluation:* performed by a neurologist, including anamnesis, physical examination, and clinical diagnosis.

*Neuropsychological evaluation:* performed by a neuropsychologist, the protocol includes the following cognitive tests and functional scales: Mini Mental State Examination,^11^ Memory Impairment Screen,^12^ Automatic reverse series (Subtest of Test Barcelona cognitive battery, Peña-Casanova, 2005),^13^ Semantic fluency task (Subtest of Test Barcelona cognitive battery, Peña-Casanova, 2005),^13^ Free and Cued Selective Reminding Test,^14^ Boston Naming Test,^15^ Trail Making Test,^16^ Blessed Dementia Rating Scale^17^ and Alzheimer’s Disease Functional Assessment and Change Scale.^18^

*CSF collection and blood sampling:* Lumbar puncture is performed in the intervertebral space L3/L4, L4/L5 or L5/S1 using a standard needle, between 8 and 11 am. Participants had fasted for at least 8h. CSF is collected into a 10 ml sterile polypropylene sterile tube (Sarstedt, Nümbrecht, Germany; cat. no. 62.610.201). Tubes are gently inverted 5 – 10 times and centrifuged at 2000g for 10 minutes at 4ºC and aliquoted in volumes of 1.8 ml into sterile polypropylene tubes (1.8 ml cryotube Thermo scientificTM NuncTM; Thermo Fisher Scientific, Waltham, MA, USA; cat. No 377267), and immediately frozen at −80°C. Blood samples are obtained the same day of the lumbar puncture and, therefore, in fasting conditions. Whole blood is drawn with a 20g or 21g needle gauge into a 10 ml ethylenediaminetetraacetic acid (EDTA) tubes (BD Vacutainer 10 ml; K2EDTA; cat. no. 367525). Tubes are gently inverted 5 – 10 times and centrifuged at 2000g for 10 minutes at (4ºC). The supernatant is aliquoted in volumes of 1.8 ml into sterile polypropylene tubes (1.8 ml cryotube Thermo scientificTM NuncTM; Thermo Fisher Scientific, Waltham, MA, USA; cat. No 377267), and immediately frozen at −80°C.

*APOE genotyping: APOE* genotyping was performed at Laboratori de Referència de Catalunya (LRC) analysing patient's genomic DNA by means of allelic discrimination's PCR assays using "APOE Real Type" reagents, from Progenie Molecular (Valencia, Spain), studying two polymorphisms: rs7412 (g8041C> T) and rs429358 (g7903 T> C) to define the *APOE* diplotypes for the ε2, ε3 and ε4 alleles, which in turn encode for the most common APOE isoforms in the population: apoE2, apoE3 and apoE4 respectively.

AD CSF core biomarkers measurements and cutoff values in the BIODEGMAR cohort

Core AD CSF biomarkers (Aβ42, Aβ40, p-tau181 and t-tau) in the BIODEGMAR cohort were measured at LRC with Lumipulse G600II (Fujirebio). The CSF sample processing protocol for these measurements at the Laboratori de referència de Catalunya (LRC) as follows: CSF is collected into a 10 ml sterile polypropylene sterile tube (Sarstedt, Nümbrecht, Germany; cat. no. 62.610.201). Tubes are gently inverted 5 – 10 times and centrifuged at 2000g for 10 minutes at room temperature (RT) and aliquoted in volumes of 0.5 ml into sterile polypropylene tubes (Sarstedt, Nümbrecht, Germany; cat. no.72.730.006) at Hospital del Mar’s laboratory and immediately shipped with dry ice to LRC, where its frozen at −80°C until measurements are performed. Cutoff values for biomarkers and ratios (Aβ42, Aβ42/Aβ40, p-tau181, t-tau-, Aβ42/p-tau181) were previously defined in the CORCOBIA study.^19^ The main goal of the CORCOBIA study was to determine the cut-off points of core AD CSF biomarkers for several centers (Parc de Salut Mar, Barcelona and Hospital General de Granollers), which work with the same reference laboratory (Laboratori de Referència de Catalunya). CSF biomarkers measurements of a group of cognitively unimpaired individuals (n = 42) and a group of mild AD dementia patients^2^ (n = 48) were compared. The cutoffs chosen were those a with higher Youden index (S+E-1). The resulting cut-offs and their AUC were the following: Aβ42 750 pg/ml (AUC 0.809); Aβ42/Aβ40 0.062 (AUC 0.78); pTau181 69.85 pg/ml (AUC 0.81); tTau 522.0 pg/ml (AUC 0.79); Aβ42/tTau 1.76 (AUC 0.86); Aβ42/pTau181 10.25 (AUC 0.86). The ratio CSF Aβ42/pTau181 shows the highest AUC and better balance between sensitivity and specificity. A more detailed description of the cohort and biomarkers measurements and cutoffs can be found in Puig-Pijoan, A, et al.
